# Use of the National Diabetes Data Group and the Carpenter-Coustan criteria for assessing gestational diabetes mellitus and risk of adverse pregnancy outcome

**DOI:** 10.1186/s12884-016-1030-9

**Published:** 2016-08-17

**Authors:** Mei-Chun Lu, Song-Shan Huang, Yuan-Horng Yan, Panchalli Wang

**Affiliations:** 1 Department of Medical Research, Kuang Tien General Hospital, Taichung, Taiwan; 2 Department of Obstetrics and Gynecology, Ditmanson Medical Foundation Chia-Yi Christian Hospital, 539 Chung-Shau Road, Chia-Yi City, 600 Taiwan; 3 Department of Internal Medicine, Kuang Tien General Hospital, Taichung, Taiwan; 4 Institute of Occupational Medicine and Industrial Hygiene, College of Public Health, National Taiwan University, Taipei, Taiwan; 5 Department of Nutrition and Institute of Biomedical Nutrition, Hung Kuang University, Taichung, Taiwan

**Keywords:** Carpenter-Coustan criteria, National Diabetes Data Group criteria, Adverse pregnancy outcome, Gestational diabetes mellitus, Macrosomia, Admission to a neonatal intensive care unit

## Abstract

**Background:**

The influence of different diagnostic thresholds for gestational diabetes mellitus (GDM) on pregnancy outcomes is not fully understood. Degrees of glucose intolerance according to the Carpenter-Coustan (CC) criteria were less severe than the National Diabetes Data Group (NDDG) criteria for GDM. Recent studies have shown inconsistent results regarding the risk of adverse pregnancy outcomes between the NDDG and CC criteria. Therefore, the objective of this study was to investigate whether pregnant women who met only the CC criteria but not the NDDG criteria and those who met the NDDG criteria had increased risks of adverse pregnancy outcomes compared to a negative screening group.

**Methods:**

A total of 11,486 Taiwanese pregnancies were enrolled in a retrospective cohort study. The study subjects were classified as follows: (1) negative screening group: women with negative 50-g glucose challenge test (GCT) results, (2) false-positive screening group: women with positive GCT results and negative 100-g OGTT results according to both CC and NDDG criteria, (3) CC-only-GDM group: women with positive GCT results plus GDM diagnosis meeting the CC but not the NDDG criteria, and (4) NDDG-GDM group: women diagnosed with GDM using the NDDG criteria. Multiple mixed effects logistic regression analysis was used to examine the relationships between the groups and pregnancy outcomes.

**Results:**

There were 9002 (78.4 %), 1776 (15.5 %), 251 (2.2 %), and 457 (4.0 %) study pregnancies in the 4 groups. Compared with the negative screening group, the maternal outcomes were not different within groups except for gestational hypertension/preeclampsia. For neonatal outcomes, the CC-only-GDM group had significantly greater risks of macrosomia, low birth weight, and admission to a neonatal intensive care unit [adjusted odds ratio (aOR), (95 % confidence interval, CI): 2.73 (1.18–6.31), 1.64 (1.01–2.64), and 1.61 (1.05–2.46), respectively]. The NDDG-GDM group also showed significantly greater risks, and the false-positive screening group showed no differences from the negative screening group.

**Conclusion:**

Women who met only the CC criteria and women who met NDDG criteria had significant increased risks of adverse neonatal outcomes. This evidence adds important information to the current debate about the diagnostic criteria for GDM regarding pregnancy outcomes.

**Electronic supplementary material:**

The online version of this article (doi:10.1186/s12884-016-1030-9) contains supplementary material, which is available to authorized users.

## Background

The prevalence of gestational diabetes mellitus (GDM) ranges between 0.7 and 10.1 % according to different diagnostic criteria worldwide [1–4]. Although the early detection of GDM is important for reducing adverse pregnancy outcome, its diagnosis remains controversial [5–8]. In 2001, the American College of Obstetricians and Gynecologists (ACOG) recommended a two-step approach: a 50-g, 1-h glucose challenge test (GCT) and a 100-g, 3-h oral glucose tolerance test (OGTT) for pregnant women with a positive GCT [9]. Two diagnostic criteria, the National Diabetes Data Group (NDDG) criteria [10] and the Carpenter-Coustan (CC) criteria [11] for the 100-g OGTT could be used. In our hospital, GDM is diagnosed according to the NDDG criteria when two or more plasma glucose levels exceed or are equal to 105, 190, 165, and 145 mg/dL for the fasting, 1-, 2- and 3-h plasma glucose tests, respectively [10]. The glucose levels described in the CC criteria are 95, 180, 155, and 140 mg/dL for the fasting, 1-, 2- and 3-h plasma glucose tests, respectively [11].

In 2010, the International Association of Diabetes and Pregnancy Study Groups (IADPSG) developed new diagnostic criteria that are based on the Hyperglycemia and Adverse Pregnancy Outcome (HAPO) [5, 6]. The ACOG subsequently reaffirmed its recommendation of the two-step approach because of concerns about a new one-step approach, which showed no evidence of improving pregnancy outcomes and would significantly increase health care costs [7, 12]. Recently, ADA indicated that GDM screening can be accomplished using either of two strategies: the one-step 2-h 75-g OGTT or the two-step approach with a 1-h 50-g GCT followed by a 3-h 100-g OGTT for those who were GCT-positive.

Despite the different recommendations for the diagnostic threshold of GDM, the two-step approach remains commonly used worldwide. However, both the NDDG and CC criteria have been used, and the ACOG has not recommended either set of criteria over the other [13]. The National Institutes of Health (NIH) consensus development conference also recommends the use of the two-step approach with a 100-g OGTT using the CC or NDDG criteria [14].

Recent studies have attempted to determine whether the use of CC criteria or NDDG criteria affect pregnancy outcome but showed inconsistent results regarding the risk of adverse pregnancy outcome between the NDDG and CC criteria [15–20]. Additional file 1: Table S1 is a brief summary of these results. Thus, it was important to determine and understand the prevalence and risk for pregnant women with GDM who were diagnosed based only on the CC criteria (not on the NDDG criteria). Therefore, our main hypothesis was that the risk of adverse pregnancy outcome in women who were diagnosed with GDM based only on the CC criteria but not meeting the NDDG criteria was significantly higher than the risk in women who screened as negative based on the GCT. The second hypothesis was that there was an increased risk of adverse pregnancy outcome in women with GDM according to the NDDG criteria and that there was no difference in women with false-positive screening compared to women with negative 50-g GCT results.

## Methods

### Study participants and data collection

This retrospective cohort study collected laboratory data and medical records from pregnant women who were administered a 50-g GCT at 24 to 28 weeks of gestation and were delivered at the Ditmanson Medical Foundation Chia-Yi Christian Hospital (DMF-CYCH) between March 2006 and January 2013. Women with multifetal pregnancies, pre-existing diabetes, and pre-existing hypertension were excluded. This study was approved by the Institutional Review Board of the DMF-CYCH (CYCH IRB No: 100006). Plasma glucose levels were measured using a Hitachi 7170 automatic analyzer (Hitachi Co., Tokyo, Japan) at the DMF-CYCH central laboratory according to a standard clinical protocol.

### Two-stage approach for GDM screening and the classification of study participants

The study included all women who underwent the two-step approach for screening and diagnosing GDM during the study period. The GCT was considered negative if the screening value was <140 mg/dL. If the GCT was positive, the women subsequently underwent a 100-g, 3-h OGTT to confirm GDM. Study subjects were classified as follows: (1) negative screening group: women with negative GCT results, (2) false-positive screening group: women with positive GCT results and negative 100-g OGTT results according to both CC and NDDG criteria, (3) CC-only-GDM group: women with positive GCT results plus GDM diagnosis meeting the CC but not the NDDG criteria, and (4) NDDG-GDM group: women diagnosed with GDM using the NDDG criteria.

### Pregnancy outcomes: neonatal and maternal outcomes

The measured pregnancy outcomes included adverse neonatal outcomes (including macrosomia (>4000 g), preterm labor (delivery before 37 weeks), low birth weight (<2500 g), admission to neonatal intensive care (NICU), and Apgar scores <7 at 1/5 min) and maternal outcomes (including cesarean section, gestational hypertension or preeclampsia, shoulder dystocia, third- or fourth-degree perineal laceration, and postpartum hemorrhage). Gestational hypertension was defined as blood pressure ≥140 mmHg systolic or ≥90 mmHg diastolic after 20 weeks of gestation in a woman with previously normal blood pressure and blood pressure levels that returned to normal postpartum. Preeclampsia was characterized by gestational hypertension and proteinuria (≥0.3 g/day or ≥1+ on a urine dipstick) with or without pathologic edema [21].

### Statistical analysis

In total, 9580 women and 11,468 pregnancies were enrolled in the study. Among the women, 1814 (18.9 %) were recruited more than once because of multi-gravidity during the study period. Therefore, the difference among the glucose level groups was evaluated using the SAS SURVEYFREQ, SURVEYMEANS and SURVEYREG procedures. Data was clustered according to subject, and the variance of proportion was estimated using the Taylor series linearization method. The Rao-Scott chi-square test was used to test the categorical variables. The Wald F test was used for continuous data. Multiple mixed effects logistic regressions were used to determine the relationships between pregnancy outcome and glucose level group after adjusting for nulliparity, maternal age, body mass index (BMI) at delivery, and delivery year. In addition to the above confounding factors, delivery type (Cesarean section or not) was added and adjusted for the maternal outcome of postpartum hemorrhage. The associations found were described in terms of the adjusted odds ratio (aOR) with a 95 % confidence interval (CI). Logistic regression is widely used to adjust for confounders, not only in case-control studies, but also in cohort studies, yielding an aOR that approximates the adjusted relative risk when the disease is rare (<10 % incidence) [22, 23]. In addition, we reviewed previous studies that are listed in the PubMed and Cochrane databases; 6 large-sample size studies reported the issue, and 4 of these articles used multiple logistic regressions to estimate risk. For comparison, we used logistic rather than Poisson regression. A two-sided *p* value < 0.05 was considered statistically significant. All data were merged and analyzed using SAS 9.2 (SAS Institute, Cary, NC, USA). Our study adheres to the STROBE (Strengthening the Reporting of Observational Studies in Epidemiology) guidelines.

## Results

**Fig. 1:**
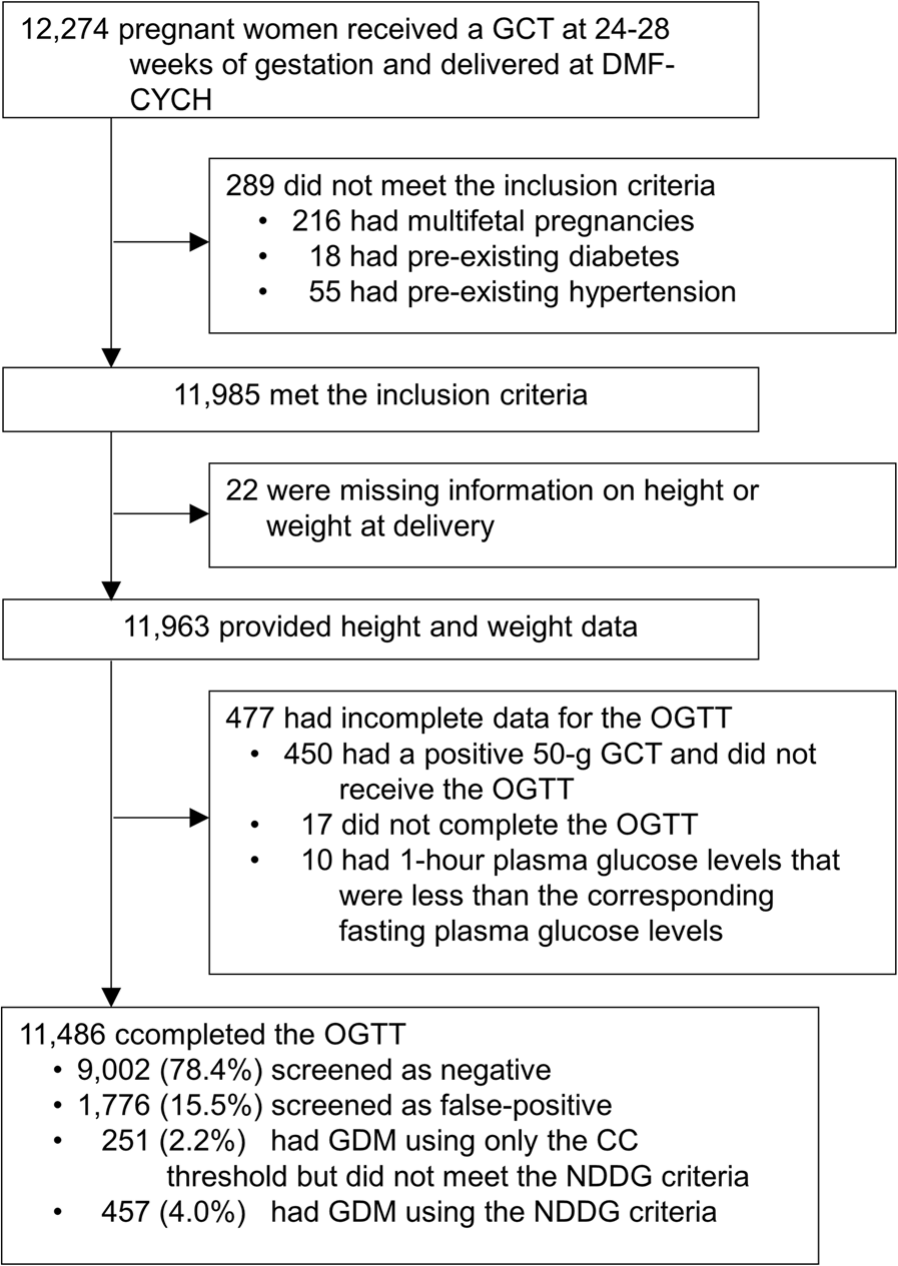
Flowchart of the study population. Footnote: GCT = glucose challenge test; DMF-CYCH = Ditmanson Medical Foundation Chia-Yi Christian Hospital; OGTT = oral glucose tolerance test; GDM = gestational diabetes mellitus; CC = Carpenter-Coustan criteria; NDDG = National Diabetes Data Group criteria

A total of 12,274 pregnancies who received a GCT at 24 to 28 weeks of gestation and who delivered at DMF-CYCH were enrolled. Among the cases, 289 women with multifetal pregnancies, pre-existing diabetes, and pre-existing hypertension were excluded; information on height or weight at delivery was missing for 22 pregnancies. An additional 477 pregnancies were excluded from the analysis due to incomplete OGTT data. Thus, a total of 11,486 pregnancies were included in the study; of these, 9002 (78.4 %) screened negative, 1776 (15.5 %) screened false-positive, 251 (2.2 %) had CC-only-GDM, and 457 (4.0 %) had NDDG-GDM (Fig. 1). The characteristics of the study population among the 4 groups are shown in Table 1. The distributions differed among these groups.

**Table 1:** Characteristics of the study population

	Negative screening	False-positive screening	CC-only-GDM (not meeting NDDG)	NDDG-GDM	*P*
Number of observations	9002	1776	251	457	
Number of clusters	7640	1699	249	434	
Nulliparous status					
Yes	4627 (51.4)	936 (52.7)	115 (45.8)	212 (46.4)	0.03^a^
No	4375 (48.6)	840 (47.3)	136 (54.2)	245 (53.6)	
Maternal age (years)	29.0 (28.9–29.1)	30.6 (30.4–30.8)	32.1 (31.5–32.6)	32.5 (32.0–32.9)	<0.001^b^
BMI at delivery (kg/m^2^)	26.7 (26.6–26.8)	27.2 (27.0–27.4)	28.2 (27.8–28.7)	28.1 (27.7–28.5)	<0.001^b^
50-g GCT levels (mg/dL)	110.4 (110.0–110.7)	158.0 (157.3–158.7)	164.8 (162.4–167.3)	179.7 (177.0–182.3)	<0.001^b^

Data are presented as means (95 % confidence interval) or *n* (%)

*CC* Carpenter-Coustan criteria, *GDM* gestational diabetes mellitus, *NDDG* National Diabetes Data Group criteria; *BMI* body mass index, *GCT* glucose challenge test

^a^The Rao-Scott chi-square test was performed using SAS SURVEYFREQ produces

^b^ANOVA was performed using SAS SURVEYREG produces the Wald F test with their corresponding *p*-values

Table 2 shows that the neonatal outcomes were significantly associated with the 4 groups, including macrosomia (0.8 to 3.5 %, *P* < 0.001), preterm labor (6.5 to 12.3 %, *P* < 0.001), low birth weight (5.6 to 9.0 %, *P* = 0.01), and NICU admission (6.5 to 12.7 %, *P* < 0.001); all of these conditions presented significantly higher incidence rates with higher glucose levels. The incidences of the maternal outcomes did not differ among the 4 glucose level groups, except for total cesareans (30.2 to 42.2 %, *P* < 0.001) and gestational hypertension or preeclampsia (2.8 to 7.0 %, *P* < 0.001), for which the incidence increased with glucose level.

**Table 2:** Pregnancy outcomes associated with the 4 glucose level groups

Variable	Number	Negative screening (*n* = 9002)	False-positive screening (*n* = 1776)	CC-only-GDM (not meeting NDDG) (*n* = 251)	NDDG-GDM (*n* = 457)	*Rao-Scott Chi-Square*	*P*
Neonatal outcome							
Macrosomia (>4000 g)	120/11,486	75 (0.8)	22 (1.2)	7 (2.8)	16 (3.5)	39.0	<0.001
Preterm labor (<37 weeks)	789/11,486	581 (6.5)	127 (7.2)	25 (10.0)	56 (12.3)	27.0	<0.001
Low birth weight (<2500 g)	673/11,486	507 (5.6)	105 (5.9)	20 (8.0)	41 (9.0)	10.9	0.01
Admission to NICU^a^	789/11,471	584 (6.5)	121 (6.8)	26 (10.4)	58 (12.7)	31.1	<0.001
Apgar score <7 at 1 min	127/11,486	99 (1.1)	17 (1.0)	4 (1.6)	7 (1.5)	1.7	0.64
Apgar score <7 at 5 min	37/11,486	28 (0.3)	8 (0.5)	-	1 (0.2)	-	-
Maternal outcome							
Cesarean section	3632/11,486	2721 (30.2)	617 (34.7)	101 (40.2)	193 (42.2)	46.3	<0.001
Gestational hypertension or preeclampsia	383/11,486	255 (2.8)	82 (4.6)	14 (5.6)	32 (7.0)	38.6	<0.001
Shoulder dystocia^b^	79/7854	64 (1.0)	7 (0.6)	3 (2.0)	5 (1.9)	5.5	0.14
Third- or fourth-degree perineal laceration^b^	429/7854	337 (5.4)	69 (6.0)	9 (6.0)	14 (5.3)	0.7	0.86
Postpartum hemorrhage	98/11,486	76 (0.8)	16 (0.9)	3 (1.2)	3 (0.7)	0.6	0.89

Data are presented as *n* (%)

*CC* Carpenter-Coustan criteria, *GDM* gestational diabetes mellitus, *NDDG* National Diabetes Data Group criteria, *NICU* neonatal intensive care unit

The Rao-Scott Chi-Square test was performed

^a^Excluding neonatal death

^b^Only including vaginal delivery

Table 3 shows the odds ratios of the outcomes after adjusting for confounding factors. Compared with the negative screening group, women with CC-only-GDM had significantly higher odds of macrosomia (aOR, 2.73; 95 % CI, 1.18–6.31), low birth weight (aOR, 1.64; 95 % CI, 1.01–2.64), and NICU admission (aOR, 1.61; 95 % CI, 1.05–2.46). The NDDG-GDM group also showed significant results with greater aOR (95 % CI): 3.15 (1.71–5.80), 1.81 (1.28–2.56), and 1.98 (1.47–2.68), respectively. The false-positive screening group did not differ from the negative screening group. Maternal outcomes did not significantly differ in the multivariable analyses, with the exceptions of gestational hypertension and preeclampsia.

**Table 3:** Adjusted odds ratios for pregnancy outcomes

Variable	Negative screening (*n* = 9002)	False-positive screening (*n* = 1776)	CC-only-GDM (not meeting NDDG) (*n* = 251)	NDDG-GDM (*n* = 457)
Neonatal outcome				
Macrosomia (>4000 g)	1	1.30(0.79–2.15)	2.73(1.18–6.31)^c^	3.15(1.71–5.80)^c^
Preterm labor (<37 weeks)	1	1.09(0.89–1.34)	1.53(0.99–2.37)	1.90(1.39–2.58)^c^
Low birth weight (<2500 g)	1	1.07(0.85–1.33)	1.64(1.01–2.64)^c^	1.81(1.28–2.56)^c^
Admission to NICU^a^	1	1.02(0.83–1.25)	1.61(1.05–2.46)^c^	1.98(1.47–2.68)^c^
Apgar score <7 at 1 min	1	0.78(0.46–1.32)	1.20(0.43–3.35)	1.13(0.51–2.51)
Maternal outcome				
Cesarean section	1	1.06(0.94–1.21)	1.11(0.82–1.50)	1.19(0.95–1.49)
Gestational hypertension or preeclampsia	1	1.36(1.04–1.79)^c^	1.42(0.79–2.58)	1.70(1.11–2.60)^c^
Shoulder dystocia^b^	1	0.61(0.27–1.34)	1.95(0.59–6.46)	2.02(0.78–5.23)
Third- or fourth-degree perineal laceration^b^	1	1.04(0.78–1.37)	1.05(0.52–2.14)	0.98(0.55–1.74)
Postpartum hemorrhage	1	1.02(0.59–1.77)	1.26(0.39–4.08)	0.68(0.21–2.21)

Data are presented as adjusted odds ratios (95 % confidence intervals)

Odds ratios were adjusted for nulliparity, maternal age, body mass index at delivery, and delivery year. In addition to the above confounding factors, delivery type (Cesarean section or not) was added for adjustment based on the perinatal outcome of postpartum hemorrhage

*CC* Carpenter-Coustan criteria, *GDM* gestational diabetes mellitus, *NDDG* National Diabetes Data Group criteria, *NICU* neonatal intensive care unit

^a^Excluding neonatal death

^b^Only including vaginal delivery

^c^95 % confidence interval did not include 1

## Discussion

Our result was concordant with other studies that use of the CC criteria of 100-g OGTT in the two-step approach increased the prevalence of GDM. Previous studies comparing the NDDG and CC criteria were conducted in the Eastern U.S. [20], Western U.S. [16, 18], Canada [15], Spain [17], Turkey [24], and Taiwan [19]. The increase in prevalence of GDM in our study was similar to that found in two large sample studies [16, 18]. The GDM prevalence increased from 31.8 to 125.7 % (Additional file 1: Table S1) using the CC criteria compared with the NDDG criteria, possibly due to differences in ethnicity, lifestyle, or policies.

The findings of the study agreed with previous studies in that the additional patients that are diagnosed by CC-only-GDM criteria are at higher risk of adverse pregnancy outcome [15, 16, 18, 20]. In addition to fetal macrosomia, the pregnant women who were diagnosed based on CC-only-GDM criteria were also at significantly increased risk of low birth weight and admission to NICU; these represent new findings (Additional file 1: Table S1). Our findings provide important additional information to the current debate about the diagnostic criteria for GDM regarding pregnancy outcome [25].

Although the incidence of macrosomia in the study was lower than that found in other studies [15–20], the rate remained significantly higher in the CC-only-GDM group than in the screening negative group. After adjusting for confounding factors, macrosomia remained a greater risk, consistent with the data reported by Berggren et al. [20]. However, a study in Spain did not yield significant findings [17].

For other neonatal outcomes, our results also showed strong evidence of significantly increased risks of low birth weight and admission NICU, and weak evidence of increased risk of preterm labor. Regarding the risk of NICU admission, two U.S. studies showed no association with the CC-only group [18, 20]; however, our data showed a significant association. Regarding low birth weight, the study of Berggren et al. showed no significant risk; however, our study showed a 1.6-fold higher risk compared to the screening negative group [20]. Regarding preterm labor, previous studies showed no significant association after adjusting for confounding factors [17, 18, 20], with the exception of Hedderson et al. [16]. Our result regarding preterm labor was similar to that reported by Hedderson et al., and the adjusted odds ratios were 1.53 (0.99–2.37) and 1.53 (1.16–2.03), respectively.

In contrast, regarding adverse maternal outcomes, our results showed that Cesarean section, gestational hypertension and preeclampsia exhibited increasing incidence as glucose levels increased from the screening negative group to the NDDG-GDM group; however, the findings did not reach statistical significance in the CC-only-GDM group after adjusting for confounding factors. These results were different than those obtained in previous studies, which showed that the incidence of cesarean section [15, 16, 18, 20], gestational hypertension, and preeclampsia [16, 17, 20] was higher in the CC-only-GDM group. We speculated that this result might have been influenced by factors such as ethnicity and the BMI variable. We used BMI at delivery to adjust the risk of pregnancy outcomes instead of the pre-pregnancy BMI, because the latter information was often missing in the database.

A similar study by Chou et al. in Taiwan enrolled 10,990 pregnancies in the urban area of northern Taiwan [19]. The prevalence of CC-only-GDM in this study and in our study was 4.4 and 2.2 %, respectively. The prevalence of NDDG-GDM in this study and in our study was 4.0 and 3.5 %, respectively. These findings demonstrated that women with GDM based on the CC criteria but not based on the NDDG criteria experienced a significant increase in macrosomia compared with women without GDM (4.5 vs. 2.3 %, *P* <0.05). Other outcomes showed negative findings. Although both studies were conducted in Taiwan, the urban area (northern Taiwan) studied by Chou was different from the area examined in our study, which enrolled pregnant women in a rural area in southern Taiwan. Environmental factors, nutritional habits and socioeconomic status are therefore potential confounding factors. Furthermore, the maternal age and gestational age at delivery in Chou’s study were different than in the current study (32.0 ± 0.4 vs. 29.5 ± 4.6 years old; 38.5 ± 0.1 vs. 38.3 ± 1.4 gestational weeks).

Recent studies have focused on the new IADPSG criteria, which have further broadened the diagnostic criteria and stimulated debate about their cost-effectiveness [24, 26–28]. The latest ACOG practice bulletin clearly supports the two-step approach because the new IADPSG criteria would significantly increase health care costs and recommends that before the testing approach and diagnostic criteria for GDM are changed, the implications of such changes should be studied [12]. We have used the two-step approach for several years, and 6.2 % of GDM according to the CC is appropriate and available for all populations that are covered by National Health Insurance. If the NDDG criteria were replaced by the CC criteria, the number of pregnant women diagnosed with GDM would increase by half, apparently increasing the burden of prenatal care. However, the higher cost of adverse neonatal outcomes on health care and society might be reduced, including long-term poor outcomes such as type 2 diabetes mellitus and the risk of childhood obesity and abnormal glucose metabolism [29–31]. The cost-effective estimation of such potential outcomes warrants further study.

The study had several limitations; first, to the study was a single-hospital retrospective study. Therefore, the results obtained for Taiwanese women in the current study should not be generalized. The existing evidence is insufficient to build consensus regarding the diagnostic criteria for GDM on pregnancy outcome [25, 32, 33]. Further prospective studies and reviews are needed. Second, it would be interesting to expand our analyses by applying the IADPSG criteria to those who screened as positive and did not meet the NDDG criteria. However, because IADPSG uses a one-step approach requiring the full OGTT for the entire study population, we have no data regarding the ideas in the current study. Despite these limitations, the large sample of Asian women involved represented a major strength of our study.

## Conclusions

Our findings suggested that diagnosing GDM according to the less strict CC criteria would detect a greater number of adverse neonatal outcomes. Women who met the CC criteria but not the NDDG criteria as well as women who met the NDDG criteria were at greater risk for macrosomia, low birth weight, and admission to the NICU compared with women who screened as negative. This evidence adds important information to the current debate regarding the diagnostic criteria for GDM on pregnancy outcomes.

## Additional file

Additional file 1: Table S1. Comparison of our results with other studies. (DOC 67 kb)

## Abbreviations

ACOG: The American College of Obstetricians and Gynecologists
BMI: Body mass index
CC: The Carpenter-Coustan criteria
CI: Confidence interval
GCT: Glucose challenge test
GDM: Gestational diabetes mellitus
HAPO: The Hyperglycemia and Adverse Pregnancy Outcome observational study
IADPSG: The International Association of Diabetes and Pregnancy Study Groups
NDDG: The National Diabetes Data Group criteria
NICU: Admission to the neonatal intensive care
OGTT: Oral glucose tolerance test
OR: Odds ratio
