# Supplementary material for: Use of the National Diabetes Data Group and the Carpenter-Coustan criteria for assessing gestational diabetes mellitus and risk of adverse pregnancy outcome
Source: BMC Pregnancy Childbirth. 2016 Aug 17;16:231. doi: 10.1186/s12884-016-1030-9 (PMC4989365; doi:10.1186/s12884-016-1030-9)
Supplement: Additional file 1: Table S1. — Comparison of our results with other studies. (DOC 67 kb) [file 12884_2016_1030_MOESM1_ESM.doc]

Table S1. Comparison of our results with other studies

| Ref. no. |  | 20 | 19 | 18 | 17 | 16 | 15 |
| --- | --- | --- | --- | --- | --- | --- | --- |
| Reference | Our study | Berggren 2011 (Eastern U.S.) | Chou 2010 (Taiwan) | Cheng 2009  (Western U.S.) | Ricart 2005 (Spain) | Hedderson 2003  (Western U.S.) | Naylor 1996 (Canada) |
| Increase in prevalence | 55.0% | 42.4% | 125.7% | 57.6% | 31.8% | 55.2% | 80.4% |
| CC-only-GDM compared with | Negative screening | False-  positive screening | False-  positive + Negative screening | False-positive + Negative screening | Negative screening | Negative screening | Negative screening |
| Study design | Retrospective | Retrospective | Retrospective | Retrospective | Prospective | Retrospective | Prospective |
| Neonatal outcomes |  |  |  |  |  |  |  |
| Macrosomia ( >4,000 g) | O | O | O |  | X | O | O |
| Macrosomia ( >4,500 g) |  |  |  | O |  |  | O |
| Preterm labor (<37 weeks) | X | X | X | X | X | O |  |
| Low birth weight (<2,500 g) | O | X |  |  |  |  |  |
| Admission to NICU | O | X |  | X |  |  |  |
| Apgar score <7 at 1 min | X |  | X |  | X |  |  |
| Maternal outcomes |  |  |  |  |  |  |  |
| Cesarean section | X | O | X | O | X | O | O |
| Gestational hypertension or preeclampsia | X |  |  |  |  | O |  |
| Gestational hypertension |  | O | X |  | O |  |  |
| Preeclampsia |  | O |  | X |  |  | X |
| Shoulder dystocia | X | X | X | O |  |  |  |
| Third- or fourth-degree perineal laceration | X | X |  | X |  |  |  |
| Postpartum hemorrhage | X |  | X | X |  |  |  |
| Adjusted OR/RR | Yes | Yes | No | Yes | Yes | Only preterm delivery | Only CS |
| Multivariate analysis | Mixed effects Logistic | Poisson | No | Logistic | Logistic | Logistic | Logistic |
| Adjustment factors | Nulliparity, maternal age, BMI at delivery, and year of delivery (with mode of delivery for postpartum hemorrhage) | Parity, maternal delivery age older than 35 years old, ethnicity, delivery year; Cesarean and operative deliveries were also controlled for prior Cesarean |  | Parity, maternal age, race or ethnicity, gestational weight gain, gestational age at delivery, year of delivery, epidural anesthesia, induction of labor | Maternal age, maternal BMI, fetal sex, gestational age, macrosomia, and gestational hypertension | Age, race, gestational hypertension/preeclampsia/eclampsia, chronic hypertension, polyhydramnios, and birth weight | Maternal age, race, parity, body mass index, history of preeclampsia, history of Cesarean delivery, gestational age, and current preeclampsia |
